# Analysis of *Thaumatotibia leucotreta* (Lepidoptera: Tortricidae: Olethreutinae) mitochondrial genomes in the context of a recent host range expansion

**DOI:** 10.1186/s12862-023-02139-5

**Published:** 2023-07-03

**Authors:** Bart T.L.H. van de Vossenberg, Tom H. van Noort, Sanne H.Z. Hooiveld-Knoppers, Lucas P. van der Gouw, Jan E.J. Mertens, Antoon J.M. Loomans

**Affiliations:** 1 Molecular Biology group, Netherlands Institute for Vectors, Invasive plants and Plant Health, NVWA, Geertjesweg 15, 6706 EA, Wageningen, the Netherlands; 2 Entomology group, Netherlands Institute for Vectors, Invasive plants and Plant Health, NVWA, Geertjesweg 15, 6706 EA, Wageningen, the Netherlands

**Keywords:** False codling moth, Rosa, Polyphagy, Nextstrain

## Abstract

**Background:**

The false codling moth (FCM), *Thaumatotibia leucotreta* (Meyrick, 1913), is a significant pest of various important economic crops and is a EU quarantine pest. In the last decade the pest has been reported on *Rosa* spp. In this study we determined whether this shift occurred within specific FCM populations across seven eastern sub-Saharan countries or whether the species opportunistically switches to this novel host as it presents itself. To achieve this, we assessed the genetic diversity of complete mitogenomes of *T. leucotreta* specimens intercepted at import and analysed potential linkages with the geographical origin and host species.

**Results:**

Genomic, geographical and host information were integrated into a *T. leucotreta* Nextstrain build which contains 95 complete mitogenomes generated from material intercepted at import between January 2013 and December 2018. Samples represented seven sub-Saharan countries and mitogenomic sequences grouped in six main clades.

**Discussion:**

If host strains of FCM would exist, specialization from a single haplotype towards the novel host is expected. Instead, we find specimens intercepted on *Rosa* spp. in all six clades. The absence of linkage between genotype and host suggests opportunistic expansion to the new host plant. This underlines risks of introducing new plant species to an area as the effect of pests already present on the new plant might be unpredictable with current knowledge.

**Supplementary Information:**

The online version contains supplementary material available at 10.1186/s12862-023-02139-5.

## Background

The false codling moth (FCM), *Thaumatotibia leucotreta* (Meyrick, 1913) (Lepidoptera: Tortricidae: Olethreutinae), described from Transvaal, Pretoria, South Africa, is a significant pest of various fruit crops and cut flowers. FCM is considered a species of high phytosanitary risk because of its polyphagous nature, moderate dispersal capabilities and wide climate tolerance. FCM is native to the Afrotropical region [1, 2] and occurs across most of sub-Saharan Africa and surrounding islands [3] along a wide range of climate zones [4]. Its climatic tolerance and dispersal capabilities are illustrated in the South-African Cape region where it was first recorded as a pest species in the Eastern Cape province in 1957 [5] after which it is thought to have extended its range into the Mediterranean climate of the Western Cape region by the 1970s [6]. In 1984, FCM was observed in Israel for the first time [7] where it has since spread across the Israeli coastal plain [8]. Many countries have listed *T. leucotreta* as quarantine pest (EPPO, 2020) and took measures to prevent the introduction and spread of the species via infested fruits, vegetables, and cut flowers or branches into their territory (EFSA, 2020). FCM was declared a quarantine pest for the European Union in 2018 [9].

The dispersal capacity of FCM in agricultural environments is usually limited to the surrounding host plants [10]. In habitats with a lower density of suitable hosts, adults have been reported to disperse over distances of several hundred meters [11]. The moth can have four to ten generations per year depending on the climatic conditions [12]. Human-assisted dispersal may currently be the greatest driver of FCM’s distribution and potential introduction as an early infestation is easily overlooked. The small, translucent, and flattened eggs are deposited directly on the surface of (ripening) fruits, petals, and sepals, and are often concolorous with their substrate [13]. Newly hatched larvae immediately bore into the fruits or (as only recently observed in rose) flower buds, leaving only a small penetration hole. Clear indications of an infestation, such as frass or discolouration, tend to become apparent during the later larval stages [14]. The final instar larva exits the host to pupate on or in the soil near the host plant, well-camouflaged inside a cocoon made up of soil particles and plant debris [15].

FCM is a known to feed on a wide range of host plants native to southern Africa, representing more than 40 plant families [16–18]. It was first recorded as a pest species on crops in the early 1920s. It attacked mostly oranges and, to a lesser extent walnuts, olives, persimmons and acorns [19]. As it appears, the polyphagous nature of the moth allowed it to gradually include various cultivated, often non-native, crops such as cotton [20], bell pepper [21], stone fruits [22, 23], avocado [24], and of more recent date (2007) also cut flowers (rose) [25, 26]. The latter is noteworthy as it is not the fruit but the flower bud that is targeted, representing a difference host stage selection. Nowadays, FCM is known to have more than 130 host plant taxa in 100 genera of plants covering 51 different plant families of both cultivated and wild species [25]. As a polyphagous species that occurs throughout large parts of the African continent [27], FCM could have developed regional host preferences, potentially having evolved into different strains or subspecies. South-African FCM populations were found to exhibit geographical population structuring; at a regional level most of the molecular variation was explained by differences between individuals and not geographical distance or host preference [28]. Indeed, results on mating preference show some premise of assortative mating among geographically distinct population [29]. However, based on mating success, FCM shows no signs of reproductive isolation in South-Africa [30].

The question remains whether populations from other African countries have developed into distinct strains and whether these are linked with changes in host plant preference (distinct host strains). With the more recent host range expansion towards *Rosa* plants, the research presented here aims to answer whether this shift occurred within specific FCM populations across the Afrotropical region or whether the species opportunistically switches to this novel host when and where it occurs. We assessed the genetic diversity of complete mitogenomes of *T. leucotreta* specimens intercepted at import to identify potential linkages with geographical origin and host species. The mitochondrial genome is frequently used in phylogenetic and population-level studies [e.g [31–33].] as it typically represents a single maternally inherited molecule with low levels of recombination while being present in high copy numbers [34, 35]. We used Nextstrain [36], a collection of open-source bio-informatic tools, to create a webtool displaying the mitogenomic diversity in context of geographic origin and host species, and end-users can interactively interrogate the data provided in the webtool. The *T. leucotreta* Nextstrain build can be accessed via https://nextstrain.nrcnvwa.nl/Tleuco/20200708.

## Results

**Assembly and annotation of*T. leucotreta*mitogenomes***De Novo* assembly of Illumina data generated for the 126 *T. leucotreta* specimens resulted in assemblies containing 146 to 1,724 scaffolds representing 0.25 to 3.24 Mbp (median_scaffolds_ = 1,248; median_assembly size_ =1.25 Mbp). Assemblies of the *T. leucotreta* specimens collectively resulted in 151,920 scaffolds of which 289 produced blastn hits with the *Helicoverpa armigera* (Hübner) mitogenome. These putative mitogenomic scaffolds ranged from 1,007 to 18,331 bp (median = 5,381), of which 33 > 10 kbp scaffolds were used to build a chimeric consensus sequence that served as reference to obtain specimen-specific mitogenomic sequences (Fig. S1). Ninety-five of the 126 specimens produced reliable contiguous mapping of Illumina reads with > 10x mean read coverage. The obtained mitogenomes ranged from 15,041 to 15,397 bp in length with 21x to 1,443x mean read coverage.

**Fig. 1:**
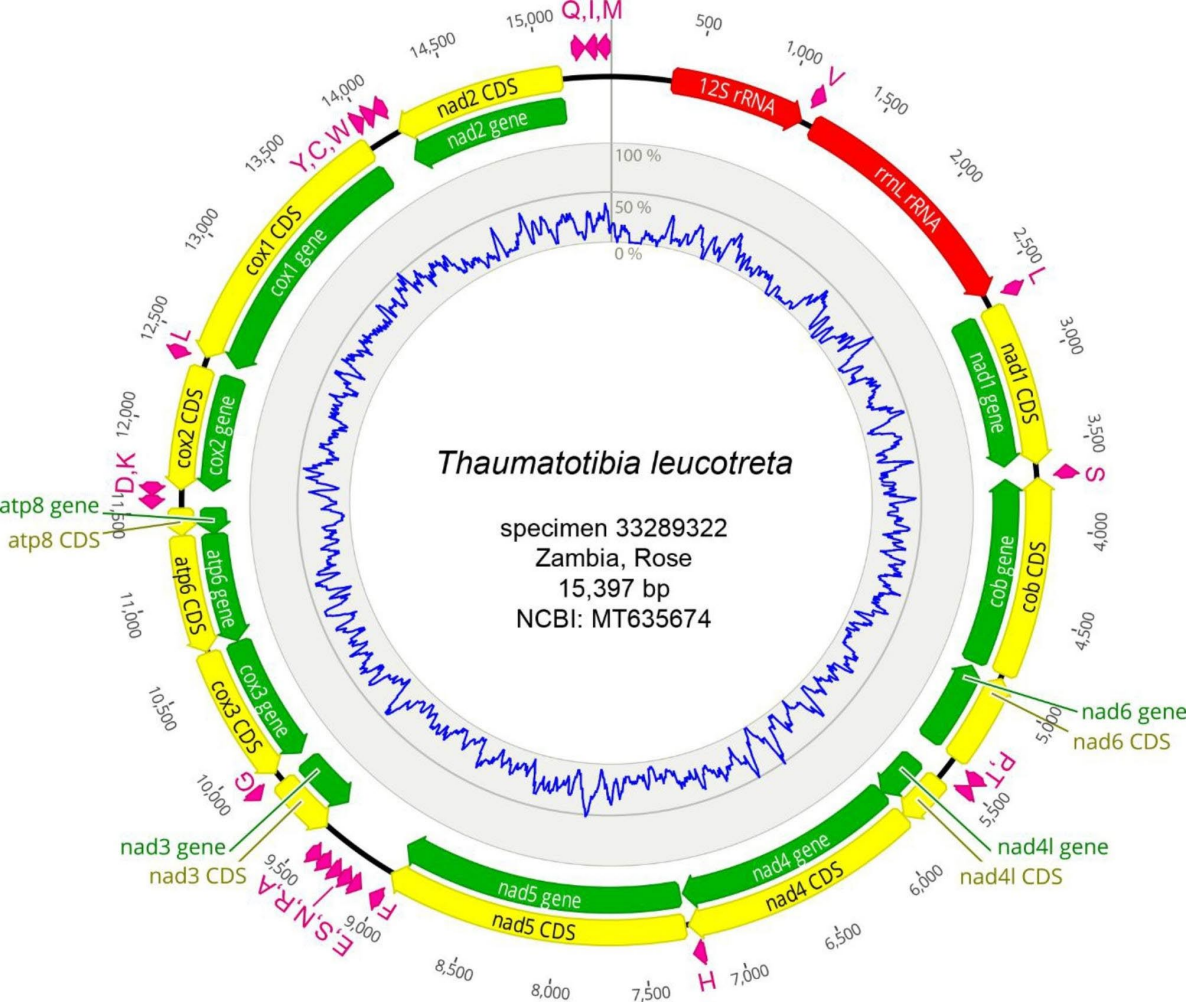
The *Thaumatotibia leucotreta* mitogenome encoding thirteen protein coding genes (annotated in green, coding sequences are annotated in yellow), twenty-two transfer RNAs (purple), and two ribosomal RNAs (red). The direction of gene transcription is indicated by the arrows and annotation lengths are proportional to their nucleotide length. Transfer RNA genes are represented by the single letter IUPAC-IUB abbreviations for their corresponding amino acid. Abbreviations for protein coding genes: atp6, atp8, ATP synthase subunits 6 and 8 genes; cob, cytochrome oxidase b gene; cox1–cox3, cytochrome oxidase c subunit 1–3 genes; nad1–6, nad4L, NADH dehydrogenase subunits 1–6 and 4 L genes

The largest mitogenomic sequence (15,397 bp) was obtained for specimen 33,289,322 which was intercepted at import in a consignment of roses from Zambia (NCBI accession MT635674). This mitogenome encoding 37 genes, which are typically found on insect mitogenomes (13 protein coding genes, 22 tRNAs, and 2 rRNAs), was used as reference for the species (Fig. 1).

Organization and orientation of the 37 mitogenomic genes were found conserved relative to publicly available complete Olethreutinae mitogenomes. In a single species (i.e. *Retinia pseudotsugaicola Liu & Wu*; NC_022865), one tRNA (tRNA-Leu) was predicted to be reverse-orientated relative to the other Olethreutinae species (Fig. S2). The “ATAGA + poly T” motif, which is conserved in lepidopteran mitogenomes and is believed to represent the origin of replication [37, 38] is located in the AT-rich control region between the tRNA-Met and 12 S rRNA genes.

**Nextstrain build** Genomic, geographical and host information were integrated in a *T. leucotreta* Nextstrain build which contains 95 complete mitogenomes generated from material sampled between January 2013 and December 2018. Information in the build is presented in three main panels: clustering of genomic diversity, geographical origin of the samples, and diversity relative to the mitogenome of specimen 33289322 (NCBI accession: MT635674). The associated metadata included in the build allows users to colour the external nodes in the tree according to the country of origin, host plant, material sampled, and the mitochondrial clade to which the specimen belongs. Internal node colours indicate the predicted ancestral state of a given trait, and the confidence of that state is conveyed by saturation of the colour of the internal node. Cladograms can be shown in different styles such as rectangular, radial and unrooted. The branch lengths of the tree can be shown based on divergence or in function of time. Based on the information provided in the build, Nextstrain determines the most likely transmission events, which can be animated from the webpage. The genotypes represented in the tree are plotted on a map, and users can set different levels of geographical resolution, i.e. continent and country. This allows simultaneous interrogation of phylogenetic and geographic relationships, with additional relevant metadata. Multiple filters can be applied to view (for instance) specific origin-host-mtDNA clade combinations. Users can download metadata and tree-files from the webpage, and can create screenshots of their views.

**Fig. 2:**
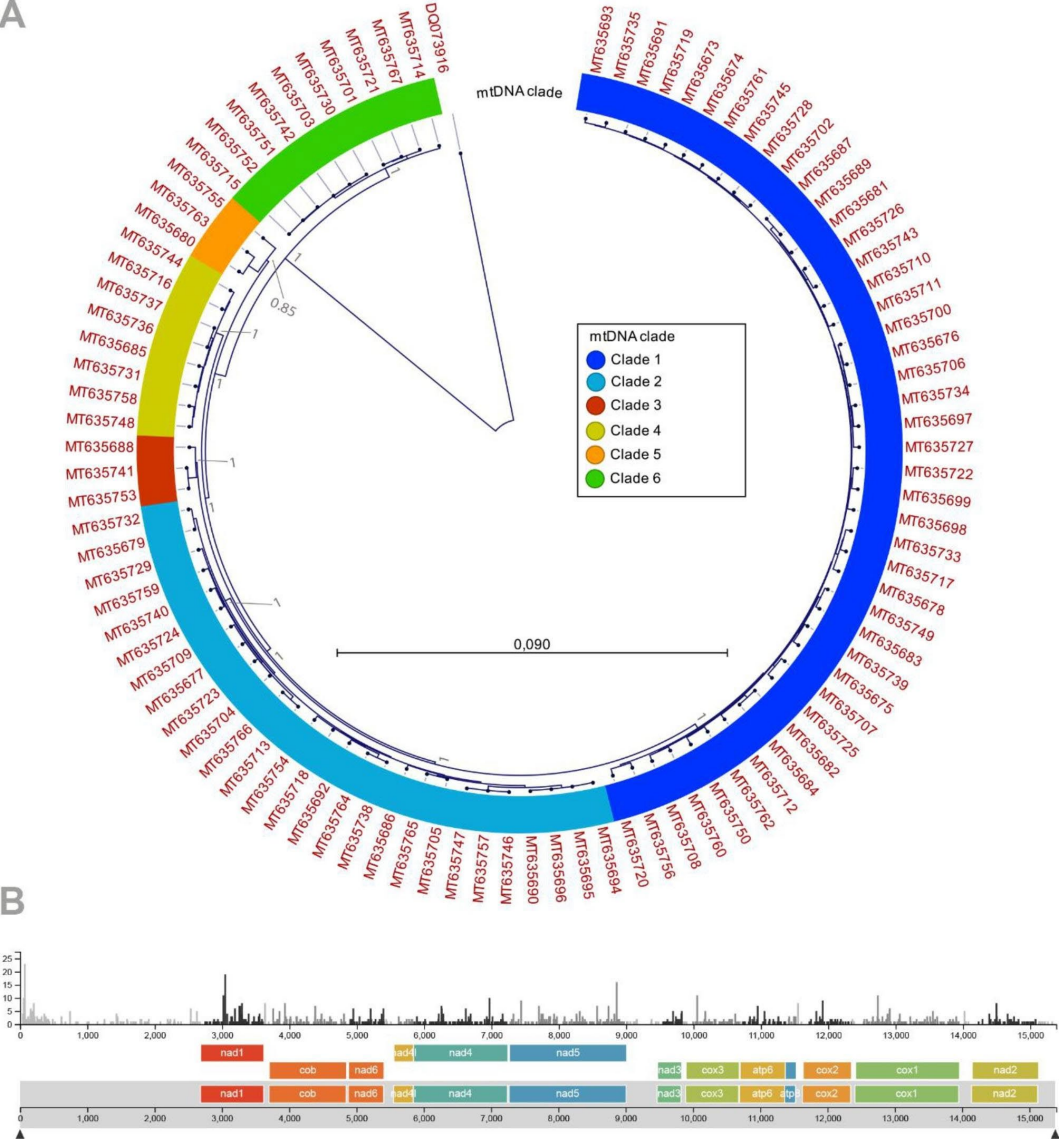
*Thaumatotibia leucotreta* intra-specific variation **(A)** MrBayes tree based on nucleotide sequences of the thirteen mitochondrial protein coding genes of 95 *T. leucotreta* specimens using *Adoxophyes honmai* as outgroup. The external codes represent the NCBI accession of the complete mitogenomic sequences from which the protein coding genes were extracted. The coloured ring indicates the major clusters (mtDNA clades) observed in the circular phylogram. Posterior probabilities are shown for the major nodes, and the scale bar represents the number of substitutions per site. **(B)** Number of single nucleotide polymorphisms per mitogenomic position relative to the 15,397 bp reference sequence (MT635674) represented in the overall *T. leucotreta* dataset. The mitogenomic diversity plot is taken from the public *T. leucotreta* Nextstrain build described in this paper

**Diversity, and host and origin associations** The mitogenomes assembled and annotated in this study are highly similar with 97.5–99.7% pairwise similarity relative to the reference sequence MT635674 (Fig. 2B). Nucleotide sequences of the thirteen protein coding genes were used to create a phylogenetic cladogram. Concatenation of the aligned gene sequences resulted in a 11,252 bp alignment with up to 116 single nucleotide polymorphisms (SNPs) among the *T. leucotreta* specimens, which equals 0–1.0% intra-species variation. Bayesian clustering of the sequences, using the leafroller species *Adoxophyes honmai* Yasuda as outgroup, results in grouping the *T. leucotreta* protein coding gene sequences in six main clades. Support for these clusters is high with 85–100% posterior probability scores for the major internal nodes (Fig. 2A). Most specimens were found to belong to mtDNA clades 1 and 2 with 44 and 27 specimens respectively. The RAxML clustering generated with entire mitogenomic sequences of the *T. leucotreta* specimens with Nextstrain is, apart from some branch swapping in the major mtDNA clades, identical to the thirteen gene Bayesian clustering.

**Fig. 3:**
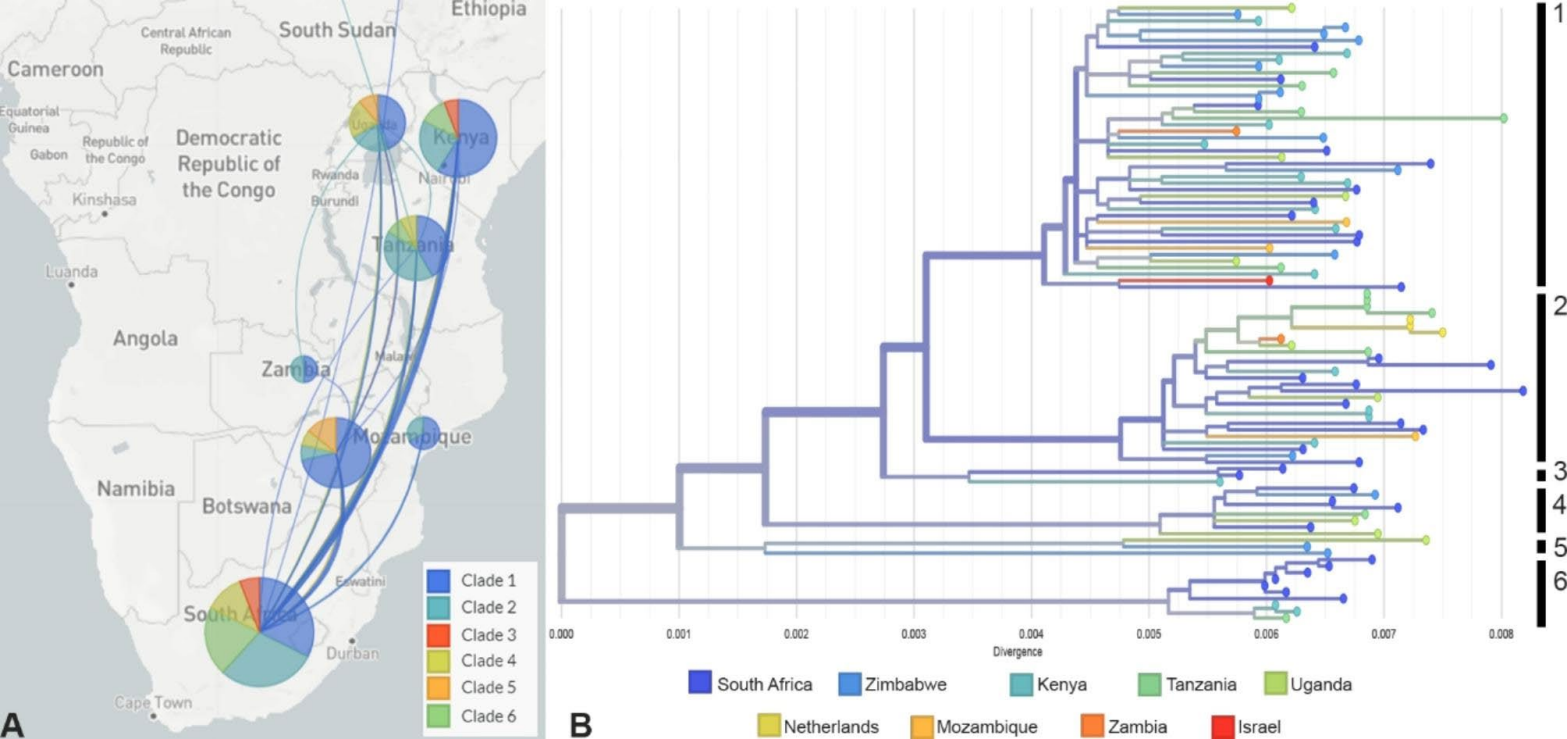
**A.** Origins of sub-Saharan *T. leucotreta* specimens in this study and mtDNA clades represented from these specimens. Bubble size indicates the number of specimens included from each country of origin. The map was generated with Mapbox, which is included in the Nextstrain tool **B.** RAxML cluster based on entire mitogenomes with external nodes coloured based on the origin of the *T. leucotreta* specimen sampled. The vertical line represents the major mtDNA clades

**Fig. 4:**
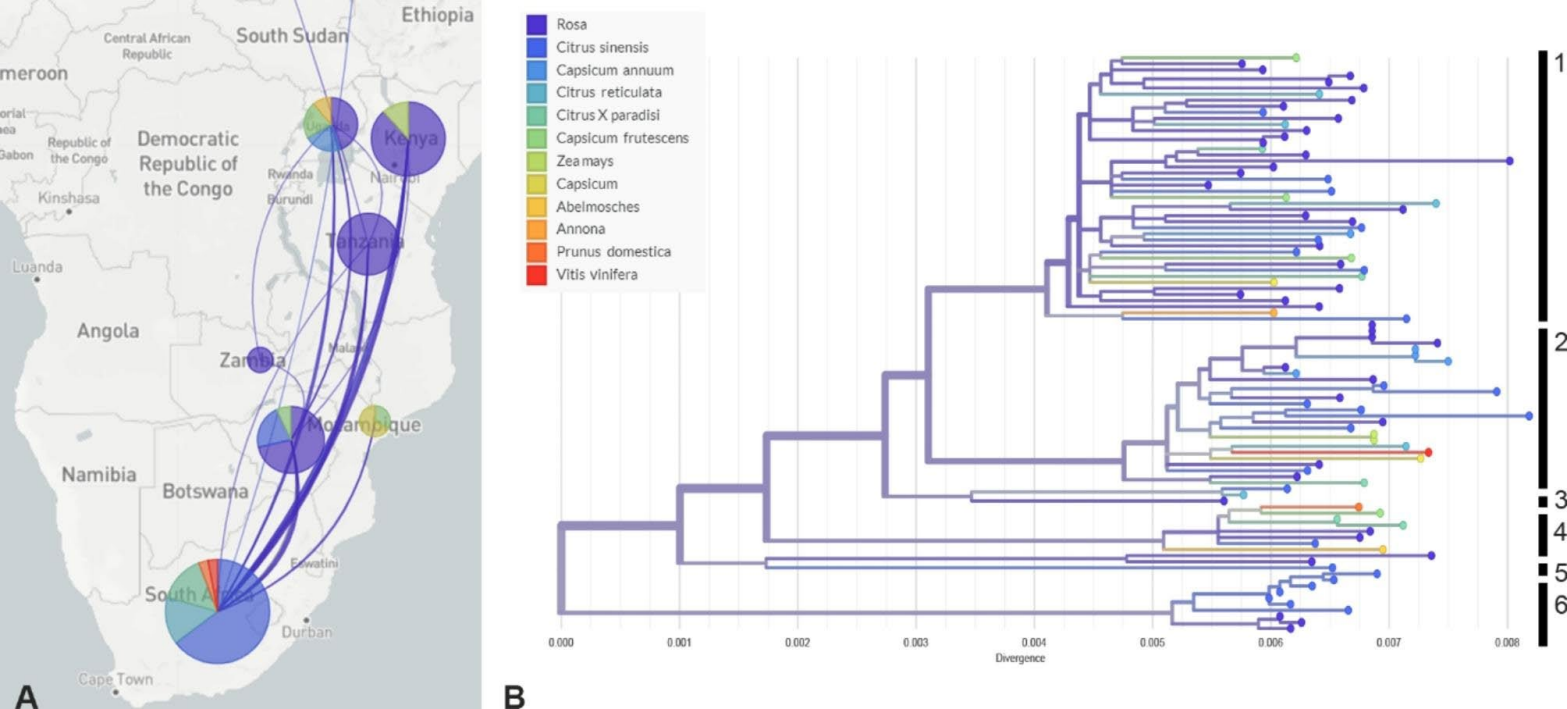
**A.** Host plants from which the *T. leucotreta* specimens were obtained from the different sub-Saharan origins included in this study. Bubble size indicates the number of specimens included from each country of origin. The map was generated with the open source Mapbox, which is included in the Nextstrain tool. **B.** RAxML cluster based on entire mitogenomes with external nodes coloured based on the host plant from which the *T. leucotreta* specimen was sampled. The vertical line represents the major mtDNA clades

Included in this study are *T. leucotreta* specimens from seven sub-Saharan African countries (Fig. 3A & B). Mitogenomic diversity observed in the overall sample set is highest in South Africa with specimens from that country being represented in all six mtDNA clades. Specimens from other sub-Saharan countries were found representing two or more mtDNA clades. The three specimens obtained at an outbreak location in the Netherlands are found in mtDNA clade 2 and are 100% identical on a nucleotide level for the thirteen protein coding genes. The specimen from Israel is found in mtDNA clade 1 and matches closest to a specimen intercepted from a *Citrus sinensis* L. x Osbeck consignment from South Africa. There are no linkages observed between mitochondrial genotype and origin of the specimens (Fig. 3B). Similarly, no linkages were observed between mitochondrial genotype and host. Specimens found on citrus fruits and rose are present in all six haplogroups (Fig. 4A), whereas specimens on *Capsicum* are found in three of the four biggest haplogroups (Fig. 4B).

## Discussion

We generated 95 complete mitogenomes of *T. leucotreta* specimens intercepted at import or sampled at outbreak locations. The import specimens represented seven sub-Saharan countries and 12 host plant species across eight genera. These sequences were used to determine the phylogenetic relationship between the specimens and to analyse potential linkages between host plant or country of origin. Based on these potential linkages we aimed to find an explanation for the occurrence of *T. leucotreta* interceptions on novel hosts, such as rose.

### Mitogenome data generation

With genome sizes in Lepidoptera species ranging from 199 Mbp to 1,253 Mbp [39], our Illumina whole genome shotgun data resulted in assemblies with median size of 1.25 Mbp representing only a small proportion of the entire *T. leucotreta* genome. However, as the mitochondrial genome is present in multiple copies per cell, the entire mitogenome could easily be assembled even when few nuclear DNA sequences were assembled. Furthermore, even when using small amounts of biological material (e.g. a single egg) as input for the sequencing, library preparation and Illumina sequencing, the complete assembled and annotated mitogenome could be obtained. Insect mitochondrial genomes are highly conserved in Lepidoptera species [40], and the *T. leucotreta* mitogenome is no exception. The 37 genes typically found in arthropods are present in the mitogenome, i.e. 13 protein coding genes, 22 transfer RNAs and 2 ribosomal RNAs, and the organization and orientation of the mitogenomic components were found conserved relative to seven publicly available complete Olethreutinae mitogenomes.

### Genetic structuring between countries

We found no proof of genetic structuring across the seven sub-Saharan countries when comparing the mitogenomes of FCM; all countries were represented by at least two different clades of FCM, with South-African interceptions spanning all six clades. The active dispersal capabilities of FCM could, in theory, be sufficient to have regular mixing of genetic material among neighbouring populations or even countries [41]. However, FCM adults do not usually disperse far when host plants are abundant [10, 42]. This is further supported by regional genetical structuring of South African FCM populations [28] and genetic variation at different altitudes in Kenya and Tanzania [43]. A study on the migratory beet armyworm (*Spodoptera exigua* (Hübner)) found little genetic structuring among the populations that migrate due to the monsoon season, whereas the clade that undergoes no or little migration had a much higher genetic differentiation and phylogeographic structure [44].

FCM is not known to show migratory behaviour. Therefore, under natural circumstances, we would expect to find a degree of genetic structuring among the countries included in this study. Instead, we find a higher genetic diversity between import consignments from within the same country of origin (e.g. all six clades in South-Africa) than those between the countries. This is in accordance with Mkiga et al. (2021) who found no genetic variability between populations in Tanzania and Kenya [43]. This absence of linkage between genotype and origin suggests that infected fruits and/or plants with soil have been transported between African countries. Citrus, as an example, has been introduced to sub-Saharan Africa on multiple occasions since late 15th century [45–47] and it is very likely to have crossed country borders from there onward, especially because of FCM’s hidden nature as larvae inside the host plant and camouflaged pupae in the soil [14, 15, 48].

### Strains linked to different host plants

Our results find no different FCM strains related to any of the 12 host plant species included in this study and *Rosa* was present as a host plant in all six clades. Moreover, specimens collected from the same host species are often less related to each other from a mitogenomic perspective than specimens collected on other hosts. The lack of linkage between genotype and plant host suggests FCM’s capability to opportunistically switch host taxa, including non-native species such as citrus in the past and *Rosa* more recently. Many factors influence whether a newly introduced plant can be a suitable host: the phenology of both parties, presence of and resistance against plant defences, landscape composition (e.g. the presence and diversity of native host plants), etc. [49–51]. Generalist and more widespread species are more likely to prefer newly introduced hosts [52] and, as a study on *Vanessa cardui* (L.) suggests, could possess adaptations that allow them to include new plant host species regardless of their phylogenetic relation to already accepted hosts [53]. FCM has many traits to its advantage: a short generation time, the possibility to disperse moderate distances to find suitable hosts, and a broad range of potential host plants in several plant families including a plethora of natural host plants. FCM has attacked new hosts on a regular basis in the past [19–25] and although its fitness varies between hosts [54], it is clearly able to sustain viable populations on cultivated hosts, possibly shifting to native hosts when needed, without having evolved specific strains related to different host plants.

Almost all samples used in this study were obtained from interceptions at Dutch import locations. Consequently, there is a sampling bias towards countries that trade large volumes of commodities on which FCM is present with the Netherlands [55]; Eastern sub-Saharan countries are well-represented, whereas Western sub-Saharan countries were absent in our samples. Even though the current study does not represent FCM’s complete distribution range, the diversity observed among the south-eastern Sub-Saharan samples is large enough to justify the determination of linkages between mitogenomic diversity and geographic origin or host on which the specimen was found.

This study showcases a unique situation where we are able to document a pest’s early inclusion of a novel host. Large-scale cultivation of cut flowers slowly shifted to tropical regions, including Africa during the late 1990s [56, 57]. Our oldest data point dates back to 2013, the first notifications of FCM on *Rosa* were made in 2007 from several African countries. If host strains of FCM would exist one would expect to find only one haplotype to be associated with this novel host. Instead, we find *Rosa* as a host in all six clades. Although our data does not focus on *Citrus* species, another important host taxon of FCM, it is likely that the association with *Citrus* and other host species is also opportunistic. It has to noted that *Prunus* spp., and thus the Rosaceae family, have been a known host of *T. leucotreta* well before the first observations on *Rosa* sp. were made [16, 58]. Taxonomically, it is not unexpected that polypgahous species can feed on other genera within an already included plant family. However, on *Prunus* spp., the eggs are laid on young fruits and the larvae will bore into the fruits to feed and develop. In *Rosa* sp., no fruit develops and the larvae feed on the flower base and petals. This illustrates the ability of the larvae to feed on plant parts or developmental stages other than the soft tissue inside fruits, which is exceptional and was previously not known for *T. leucotreta*. This stipulates the risks of introducing new crop species to an area; the effect an already present polyphagous pest on the new crop might be unpredicted with current knowledge.

## Methods

### Insect specimens

A total of 126 *T. leucotreta* samples obtained from regular import inspections performed by the Dutch National Plant Protection Organization (NPPO-NL) from January 2013 to December 2018 were included in this study. Larvae and adults were identified as *T. leucotreta* using an identification key described by Gilligan and Epstein [12] also as implemented in EPPO standard PM7/137 (1) [26]. For selected specimens Sanger Sequencing of the partial mitochondrial *cox1* gene using primers LCO1490/HCO2198 according to the EPPO DNA Barcoding standard PM7/129(1) [59] was performed in support of species identification. Alternatively, eggs were identified as cf. *T. leucotreta* based on host plant and origin association. Specimens mostly originated from interceptions from sub-Saharan Africa, i.e. South Africa: 34; Kenya: 17; Zimbabwe: 14; Tanzania: 12; Uganda: 9; Mozambique: 3; and Zambia: 2. In addition, one specimen from Israel and 3 specimens from an outbreak location in the Netherlands were included. Host plants from which the specimens were obtained belonged to several plant genera, i.e. *Rosa*: 43; *Citrus*: 35; *Capsicum*: 11; *Zea*: 2; *Abelmosches*: 1; *Annona*: 1; *Prunus*: 1; and *Vitis*: 1. An overview of specimen information and associated molecular data is provided in Table S1.

### DNA extraction and Illumina sequencing

Single eggs, larvae and parts of adult specimens were used as input for the DNA extraction. Insect tissue was ground in a lysis buffer with a micro-pestle prior to DNA extraction with the QuickPick SML genomic DNA kit (Bio-Nobile, Finland) on a KingFisher 96 flex (ThermoFisher, MA, USA). Genomic DNA was eluted in 100 µL elution buffer, and aliquots were sent to GenomeScan (Leiden, the Netherlands) for 150PE Illumina sequencing with the NextSeq 500 V2 platform. In short, after fragmentation of the genomic DNA with the Bioruptor Pico (Diagenode, Belgium), sequencing libraries were prepared with the NEBNext Ultra DNA Library Prep kit for Illumina following manufacturer’s instructions. Each sample was tagged to create an unique index-flowcell combination, and libraries were split over four sequencing lanes resulting in at least 2 Gb output per sample.

### Mitogenome assembly and annotation

Illumina sequence reads were uploaded to CLC genomics workbench v11 and subjected to a pipeline that combined *de novo* assembly and blast-based identification of putative mitochondrial contigs. The pipeline consisted of the following analysis steps: trim reads (Quality trim: 0.05; Ambiguous limit: 2), *de novo* assembly (mapping mode: map reads back to contigs; update contigs: ON; length fraction: 0.8; similarity fraction: 0.9; minimum length: 1 kbp), extract consensus (threshold: 10; low coverage handling: split contigs), a size selector to discard consensus sequences shorter than 500 bp, and a blastn analysis of consensus sequences to the complete *Helicoverpa armigera* mitogenome (GU188273). Consensus sequences with an E-value < 1e-50 were regarded putative mitogenomic sequences. Putative mtDNA contigs > 10 kbp were used to create a single (chimeric) circular consensus mtDNA sequence in Geneious v11 (Biomatters, New Zealand). This was achieved by iteratively mapping putative mtDNA contigs to the largest scaffold, which allowed the identification and removal of non-*T. leucotreta* sequences. Sequences that wrapped around the 5’ and 3’ end of the largest scaffold were used to identify circularity of the mitogenome. Mitochondrial sequences for the individual *T. leucotreta* specimens were obtained by mapping reads of these specimens to the circular consensus mtDNA sequence (CLC Genomics workbench, length fraction: 0.8, similarity fraction: 0.9). Specimens with ≤ 10x mean read coverage were excluded from the analysis, resulting in 95 specimens which were used in the phylogenetic and genotype association analyses. The 95 circular mitogenomes were annotated using the online MITOS v2 tool using the invertebrate mitochondrial code [60], and protein coding sequence annotations were manually checked to determine the reliability of the automated annotation. The annotated *T. leucotreta* mitogenomes were submitted in NCBI under accessions MT635673 - MT635767. The organization and orientation of *T. leucotreta* mtDNA genes was compared with that of other publicly available Olethreutinae mitogenomes using the MAUVE (version: 2015-02-26; algorithm: progressive) genome alignment tool [61] incorporated in Geneious v11, i.e.: *Celypha flavipalpana* H.-F. (NC_046051); *Cydia pomonella* (L.) (NC_020003); *Grapholita dimorpha* Komai (NC_024582); *Grapholita molesta* (Busck) (NC_014806); *Retinia pseudotsugaicola* (NC_022865); *Rhyacionia leptotubula* Liu & Bai (NC_019619); *Spilonota lechriaspis* Meyrick (NC_014294).

### Nextstrain build

To identify potential links between genotypes and geographical origin or host plants, a Nextstrain build was created based on the complete mitogenomic sequences of the 95 *T. leucotreta* specimens. Augur (github.com/Nextstrain/augur), a bioinformatics toolkit for phylogenetic analysis used in the Nextstrain pipeline, was used to align the mitogenomic sequences with MAFFT [62] and to perform a RAxML clustering [63]. The initial tree was refined with metadata and a time tree was built using TreeTime [36] with optimization of scalar coalescent time, and using a fixed clock rate of 2.3 × 10^− 8^ following Brower [64]. Internal nodes were assigned to their marginally most likely dates, and confidence intervals for node dates were estimated. Amino acid changes were determined based on the longest annotated *T. leucotreta* mitogenome, i.e. MT635674: 15,397 bp, specimen 33289322. Next, Augur output was exported and visualized in auspice. The *T. leucotreta* Nextstrain build is deposited on GitHub (https://github.com/NPPO-NL/nextstrain-Tleuco).

### Clustering analysis

Sequences of the 13 protein coding genes were extracted from the mitogenome of each of the 95 specimens. Per gene a MAFFT v7.388 [62] alignment (algorithm: FFT-NS-1) was created using gene sequences from the *Adoxophyes honmai* (Lepidoptera: Tortricidae: Tortricinae) mitogenome (DQ073916 = NC_008141) [65] as outgroup. The resulting thirteen alignments were concatenated and a Bayesian Inference of phylogeny was constructed with MrBayes v 3.2.6 [66] in Geneious v11 using the optimal substitution model as defined in the model testing module of CLC genomics workbench (i.e. JC69 : nst = 1; rate variation = equal). A total of 1 × 10^6^ replications were run, and 10% was discarded as burn-in. Trees were sampled every 1,000 replications, and the consensus tree was exported to CLC genomics workbench to colour nodes according to the mtDNA clade to which they belonged. Posterior probability values were manually added in Adobe Illustrator.

## Electronic supplementary material

Below is the link to the electronic supplementary material.

Supplementary Material 1

Supplementary Material 2

## Data Availability

The annotated T. leucotreta mitogenomes were submitted in NCBI under accessions MT635673 - MT635767. Sequence read archives 31054601 to 31054524 are submitted to NCBI under BioProject PRJNA938910.

## Abbreviations

EFSA: European Food Safety Authority
EPPO: European and Mediterranean Plant Protection Organization
FCM: False Codling Moth
NVWA: Netherlands Food and Consumer Product Safety Authority
USDA-APHIS-PPQ: The United States Department of Agriculture - Animal and Plant Health Inspection Service - Plant Protection and Quarantine
