# Supplementary material for: Analysis of Thaumatotibia leucotreta (Lepidoptera: Tortricidae: Olethreutinae) mitochondrial genomes in the context of a recent host range expansion
Source: BMC Ecol Evol. 2023 Jul 3;23:28. doi: 10.1186/s12862-023-02139-5 (PMC10316639; doi:10.1186/s12862-023-02139-5)
Supplement: Supplementary file 2 — Supplementary Material 2 [file 12862_2023_2139_MOESM2_ESM.pdf]

Supplementary figures with

Analysis of *Thaumatotibia leucotreta* (Lepidoptera; Tortricidae: Olethreutinae) mitochondrial genomes reveal opportunistic host range expansion towards rose

Bart T.L.H. van de Vossenberg[1#], Tom H. van Noort[2#*], Sanne H.Z. Hooiveld-Knoppers[1,2], Lucas P. van der Gouw[1], Jan E.J. Mertens[2], Antoon J.M. Loomans[2]

1. Netherlands Institute for Vectors, Invasive plants and Plant Health, Molecular Biology group, Geertjesweg 15, 6706EA, Wageningen, the Netherlands
2. Netherlands Institute for Vectors, Invasive plants and Plant Health, Entomology group, Geertjesweg 15, 6706EA, Wageningen, the Netherlands

# These authors contributed equally

* Corresponding author: Tom H. van Noort, t.h.vannoort@nvwa.nl

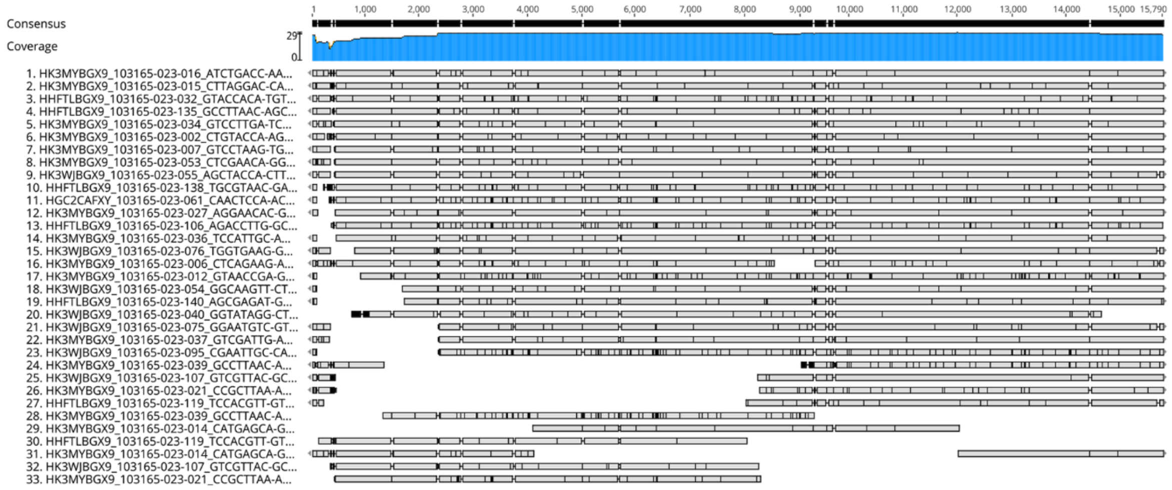

Figure S1. Reference based assembly with putative mitochondrial sequences to create a single chimeric consensus for the *T. leucotreta* mitogenome. Mismatches to the overall consensus are highlighted in black. When sequences wrap around the 5’ or 3’ end of the reference (i.e. the largest sequence in this assembly), this is indicated with a grey arrow.

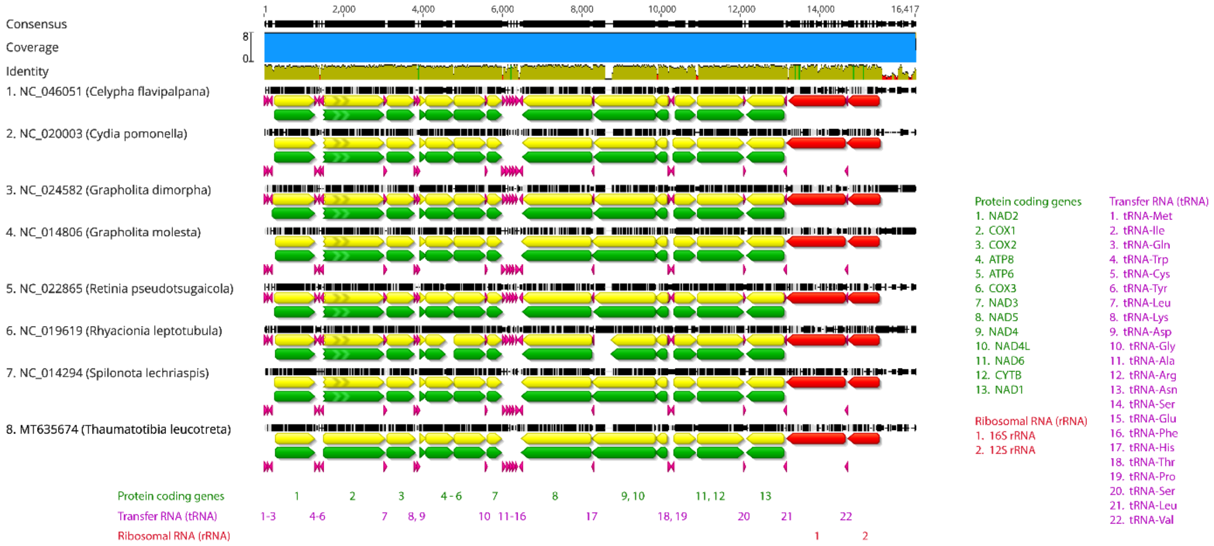

Figure S2. MAFFT alignment of structurally and functionally annotated public Olethreutinae mitogenomes. Apart from small differences in predicted start and stop codon positions for protein coding genes, and the orientation of a single tRNA in *Rhyacionia leptotubula* (tRNA-LEU, number 21 in the figure) the organization of the 37 mitogenomic elements is conserved within Olethreutinae.
